# Elderly Patients With Inflammatory Polyarthritis: The Importance of Including Remitting Seronegative Symmetrical Synovitis With Pitting Edema (RS3PE) in the Differential Diagnosis

**DOI:** 10.7759/cureus.81296

**Published:** 2025-03-27

**Authors:** Erin A Cook, Matthew C Mulroy, Benedict K Tiong

**Affiliations:** 1 Division of Geriatrics, Department of Medicine, University of California Los Angeles David Geffen School of Medicine, Los Angeles, USA; 2 Division of Rheumatology, Department of Medicine, University of California Los Angeles David Geffen School of Medicine, Los Angeles, USA

**Keywords:** erythocyte sedimentation rate, geriatrics population, goals of care, hand edema, rs3pe, screening for cancer, seronegative inflammatory arthritis, symmetric polyarthritis

## Abstract

Remitting symmetrical seronegative synovitis with pitting edema (RS3PE) is a rare seronegative inflammatory arthritis that primarily affects older adults. Clinical features include acute onset, symmetric polyarthritis in the upper extremities, pitting edema in the dorsum of the hands, age of onset > 60 years, and rapid response to medium-dose steroids. Rheumatoid factor and joint erosions are typically absent. Despite its favorable prognosis, RS3PE is frequently misdiagnosed in older adults due to overlapping comorbidities, posing diagnostic challenges. We present a case of a 92-year-old male with recurrent RS3PE who initially presented with acute symmetric upper extremity edema, polyarthritis, and a dramatic response to prednisone. His symptoms recurred three years after remission, which is unusual. As the population ages and life expectancy increases, it is likely clinicians will see an increase in the incidence of RS3PE. Understanding the clinical nuances and subtleties in distinguishing this condition from other inflammatory and crystalline arthropathies will lead to improved diagnostic accuracy and management. Furthermore, the high association of RS3PE with concurrent malignancy warrants age-appropriate cancer screening and a careful balance of diagnostic investigation and goals of care in those with advanced age. Exploration of “what matters most,” one of the pillars of age-friendly care, should guide clinicians on how best to proceed.

## Introduction

Polyarthritis is a common finding in geriatric patients, characterized by the presence of arthritis in five or more joints [1]. More than 50% of adults over age 75 have arthritis according to data from the 2022 National Health Interview Survey [2]. The most common polyarthritis is osteoarthritis, affecting one in three people over age 65 [3]. Osteoarthritis is a degenerative arthritis from the breakdown of cartilage and is different from inflammatory arthritis that results in joint destruction from inflammation and synovitis from an overactive immune system. The most common inflammatory arthritis conditions seen in older adults are rheumatoid arthritis (RA) and polymyalgia rheumatica (PMR), with the former usually diagnosed between 30 and 50 years of age and the latter presenting more commonly after the age of 65. The prevalence of RA in the population over 60 years of age is estimated to be 2%, and there is a late onset version of the disease [4]. As the population ages, clinicians will be seeing more complaints of joint pain and inflammation. There is a wide differential diagnosis for new onset polyarthritis in an older adult. The differential includes osteoarthritis, late onset RA, PMR, polyarticular crystalline arthropathies, infectious diseases, malignancy, vasculitis, connective tissue disease, sarcoidosis, and adverse effects of medications [5]. A more unusual cause of polyarthritis in older adults is remitting symmetrical seronegative synovitis with pitting edema (RS3PE), which is a seronegative inflammatory arthritis. RS3PE responds well to treatment and is associated with underlying malignancy [5]. This case report explores a classic presentation of RS3PE in an older adult with multiple comorbidities. It highlights the importance of understanding the clinical nuances in distinguishing this condition from other rheumatologic conditions. With prompt diagnosis and treatment, clinical outcomes are excellent. Furthermore, the high association of RS3PE with concurrent malignancy adds gravity to the importance of diagnostic accuracy and early detection.

## Case presentation

A 92-year-old male with a history of metastatic colon cancer, prostate cancer, congestive heart failure with preserved ejection fraction, atrial fibrillation, chronic kidney disease, amiodarone-induced hypothyroidism, osteoarthritis, and osteoporosis presented to the clinic with five weeks of bilateral hand and upper extremity swelling associated with bilateral shoulder stiffness and reduced range of motion of the affected joints. Pitting edema was noted over his bilateral hands and wrists. The patient denied injury or fall. There was no associated fever, erythema, rash, or warmth of the skin. Radiographs of the shoulders showed signs of chronic rotator cuff pathology and mild osteoarthritis. Laboratory tests taken at the time of presentation are provided in Table 1. A trial of colchicine was started for a possible diagnosis of calcium pyrophosphate deposition disease (CPPD), often referred to as pseudogout. Colchicine only provided minimal improvement of his symptoms. Due to the marked elevation in inflammatory markers, negative rheumatoid factor (RF) and anti-cyclic citrullinated peptide antibodies (ACPA), symmetric polyarticular involvement, and his history of cancer, there was concern that the patient had remitting RS3PE, and prednisone 15 mg daily was started. He was also referred to rheumatology for further evaluation.

**Table 1:** Labs at Initial Presentation Ab, antibody; IgG, immunoglobulin G; BNP, brain natriuretic peptide; TSH, thyroid-stimulating hormone; T4, thyroxine; Spec, specific

Lab	Value	Reference Range
Sedimentation rate, erythrocyte	126 mm/hr	<12 mm/hr
C-reactive protein	8.0 mg/dL	<0.8 mg/dL
Uric acid	7.4 mg/dL	3.4–8.8 mg/dL
Cyclic citrulline Ab IgG	2 units	0–19 units
Rheumatoid factor	<10 IU/mL	<25 IU/mL
Antinuclear Ab	<1:40 titer	<1:40 titer
Creatinine	1.19 mg/dL	0.6–1.3 mg/dL
Albumin	3.5 g/dL	3.9–5.0 g/dL
Calcium	8.5 mg/dL	8.6–10.4 mg/dL
Alkaline phosphatase	651 U/L	37–113 U/L
BNP	181 pg/mL	<100 pg/mL
TSH	6.3 mcIU/mL	0.3–4.7 mcIU/mL
Free T4	1.2 ng/dL	0.8–1.7 ng/dL
Alkaline phosphatase, bone spec	68.2 ug/L	6.5–20.1 ug/L
White blood cell count	7.99x10^3^/uL	4.16–9.95x10^3^/uL
Hemoglobin	9.1 g/dL	13.5–17.1 g/dL
Hematocrit	30.1%	38.5–52.0%
Mean corpuscular volume	83.8 fL	79.3–98.6 fL
Platelet count, auto	419x10^3^/uL	143–393x10^3^/uL

The patient reported a “remarkable” response to prednisone with immediate resolution of swelling in his hands and arms. He saw the rheumatologist a week after starting prednisone and the rheumatologist confirmed the diagnosis of RS3PE and planned to manage his symptoms with a three-month prednisone taper. Repeat laboratory tests (Table 2) showed a dramatic improvement of inflammatory markers after two weeks of prednisone. Due to the association of RS3PE with underlying cancer, the patient was advised to see his primary oncologist. A computed tomography (CT) scan of the abdomen/pelvis was recommended but ultimately not pursued by the patient due to his goals of care and advanced age. He underwent a radionuclide whole body bone scan due to the elevation in alkaline phosphatase levels, which showed no scintigraphic evidence for osseous metastasis. He required a longer prednisone taper due to recurrence of symptoms with lower doses and was eventually weaned off prednisone after eight months with resolution of symptoms and normalization of inflammatory markers.

**Table 2:** Labs two weeks after presentation PSA, prostate-specific antigen; CEA, carcinoembryonic antigen

Labs	Value	Reference Range
Sedimentation rate, erythrocyte	59 mm/hr	<12 mm/hr
C-reactive protein	<0.8 mg/dL	<0.8 mg/dL
Creatine phosphokinase, total	23 U/L	63–473 U/L
PSA, total	0.48 ng/mL	0.0–6.5 ng/mL
Creatinine	1.22 mg/dL	0.6–1.3 mg/dL
Albumin	3.7 g/dL	3.9–5.0 g/dL
Calcium	8.6 mg/dL	8.6–10.4 mg/dL
Alkaline phosphatase	359 u/L	37–113 U/L
CEA	4.6 ng/mL	< 3.1 ng/mL

This patient’s oncologic history is notable for multiple malignancies. At the age of 76, he was diagnosed with prostate adenocarcinoma and treated with a radical prostatectomy and androgen deprivation therapy without recurrence. Ten years later, he was diagnosed with low-grade colorectal adenocarcinoma (T3N1cM1) and underwent a right hemicolectomy followed by a hepatic wedge resection and five cycles of adjuvant capecitabine/oxaliplatin for an isolated liver metastasis. He was unable to tolerate further chemotherapy due to neuropathy. During this time, he was also found to have a pancreatic ampullary adenoma and underwent resection and biliary stent placement. He had no recurrence of disease up until the time of his above presentation.

**Figure 1:**
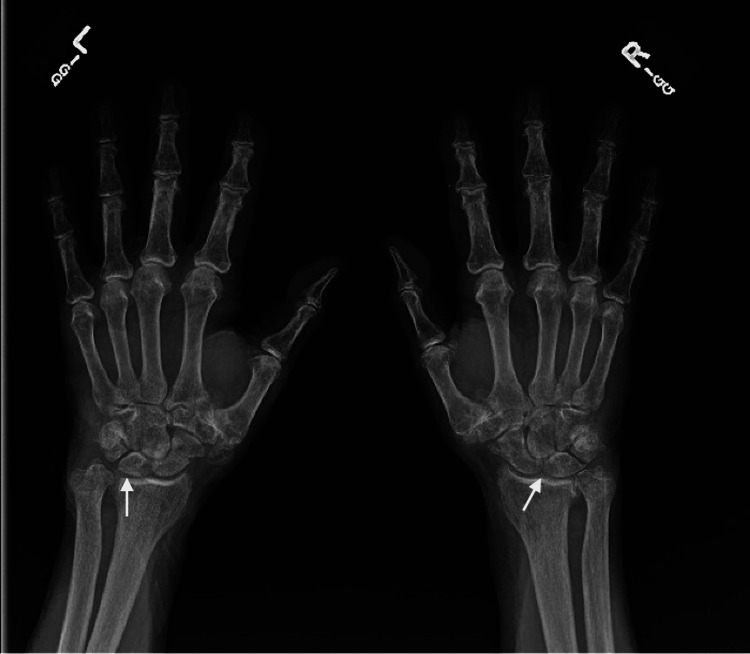
Plain radiograph of the hands Plain radiograph of the hands shows diffuse osteoarthritis and radiocarpal chondrocalcinosis (noted by arrows)

Three and half years later at the age of 95, the patient presented again to the clinic with acute onset bilateral hand pain, swelling, and erythema affecting multiple joints in fingers and wrist. Again, there was no history of trauma or falls. He was recently started on acetazolamide, and thus there was concern for an atypical presentation of a crystalline arthropathy versus recurrence of RS3PE. He was given triamcinolone 40 mg intramuscularly in the clinic and started on prednisone 40 mg daily. He also received an antibiotic to cover for possible cellulitis. A radiograph of the hands showed diffuse osteoarthritis, radiocarpal chondrocalcinosis, and lack of joint erosions (Figure 1). Labs at this visit are noted in Table 3. Again, he experienced immediate improvement of symptoms with prednisone. After three days, he was transitioned to prednisone 15 mg daily, which was tapered over six weeks.

**Table 3:** Labs during recurrence (3.5 years later) Ab, antibody; IgG, immunoglobulin G; HLA, human leukocyte antigen; C-ANCA, cytoplasmic anti-neutrophil cytoplasmic antibody; P-ANCA, perinuclear anti-neutrophil cytoplasmic antibody

Lab	Value	Reference Range
Sedimentation rate, erythrocyte	20 mm/hr	<12 mm/hr
C-reactive protein	<0.3 mg/dL	<0.8 mg/dL
Cyclic citrulline Ab IgG	2 units	0–19 units
Rheumatoid factor	<10 IU/mL	<25 IU/mL
Antinuclear Ab	<1:40 titer	<1:40 titer
Creatinine	1.96 mg/dL	0.6–1.3 mg/dL
Albumin	3.6 g/dL	3.9–5.0 g/dL
Calcium	9.0 mg/dL	8.6–10.4 mg/dL
Alkaline phosphatase	138 U/L	37–113 U/L
HLA-B27	Not present	
C-ANCA	<1:20 titer	<1:20 titer
P-ANCA	<1:20 titer	<1:20 titer
Myeloperoxidase Ab	<20 titer	<20 CU
Proteinase-3 Ab	<20 titer	<20 CU
White blood cell count	6.43 x 10^3^/uL	4.16–9.95 x 10^3^/uL
Hemoglobin	10 g/dL	13.5–17.1 g/dL
Hematocrit	34.2%	38.5–52.0%
Mean corpuscular volume	97.2 fL	79.3–98.6 fL
Platelet count, auto	231 x 10^3^/uL	143–393 x 10^3^/uL

## Discussion

RS3PE is an uncommonly diagnosed inflammatory arthritis that occurs in older adults. The incidence is not fully known due to diagnostic challenges, but one Japanese study estimated it to be 0.09% in patients over the age of 50 [6]. The disease has a 63% male predominance, with an average of onset of 71 years based on a large systemic review and meta-analysis [7,8]. RS3PE is often misdiagnosed as late onset RA or PMR and was first described in 1985 with a case series that initially characterized the disease as a subset of late onset seronegative RA [9]. It is now considered its own clinical entity. The etiology of RS3PE is undetermined but likely an autoinflammatory or autoimmune process [10]. Some postulate it could be part of a paraneoplastic syndrome or triggered by an alpha-tumor necrosis factor (TNF) release from a tumor or that there may be a genetic predisposition or infectious trigger [11,12]. Finally, RS3PE may be an adverse event of a medication as several case reports suggest dipeptidyl peptidase-4 (DPP-4) inhibitors trigger the disease [13,14]. The patient in this case was not taking a DDP-4 inhibitor.

Typical laboratory findings included a normal white blood cell count, negative RF, antinuclear antibody (ANA), and anti-citrullinated protein antibody, and elevated erythrocyte sedimentation rate (ESR) and C-reactive protein. There may be an association with human leukocyte antigen (HLA) haplotypes [10]. Joint erosions are not commonly seen on radiographs. Evidence of extensor tenosynovitis is frequently identified on ultrasound or magnetic resonance imaging (MRI). Clinical features include acute onset, bilateral symptoms, pitting edema in the dorsum of the hands (though feet can also be affected), age of onset > 60 years, and rapid response to steroids [7,8]. A low-to-medium dose of steroids was found to be effective in most case reports. RS3PE may be difficult to distinguish from PMR, but distinguishing features include male predominance, edema, and involvement of small joints over proximal joints [11,15]. It can also be distinguished from crystalline arthropathy due to the polyarticular and bilateral presentation.

In this patient, it was difficult to differentiate if his second episode of bilateral hand swelling and edema was due to an RS3PE recurrence or an acute calcium pyrophosphate deposition disease (CPPD) flare given he had radiographic findings of radiocarpal chondrocalcinosis. Both diseases present acutely and can have elevation in ESR, but RS3PE was felt to be more likely as this patient had no prior history of CPPD and had bilateral and polyarticular joint involvement of the metacarpophalangeal (MCP) joints, proximal interphalangeal (PIP) joints, and distal interphalangeal (DIP) joints in addition to the radiocarpal joints. CPPD affects the knees in more than 50% of acute presentations and the wrists, shoulders, ankles, feet, and elbows are other commonly affected joints. Small joint involvement, including MCP, PIP, and DIP joints, is not typical. Finally, RS3PE was the favored diagnosis due to his extensive malignancy history. Arthrocentesis and synovial fluid analysis of the affected joints should have been performed to further clarify the diagnosis and is recommended when crystalline arthropathy is strongly considered in the differential diagnosis. Corticosteroids are also the mainstay of treatment for CPPD, and thus there is considerable overlap in disease presentation and treatment of these two conditions.

RS3PE is associated with an underlying malignancy in 16% to 30% of cases [8,16]. Solid organ gastrointestinal and genitourinary malignancies were reported the most. Any patient with RS3PE should undergo age-appropriate cancer screening. Thorough evaluation for an underlying malignancy should only be pursued in select patients where a diagnostic workup is within their goals of care and for those who have an appropriate life expectancy and health status to benefit from cancer treatment. In this case, the patient’s advanced age, serious comorbidities, and personal preferences precluded him from an extensive malignancy workup.

Most patients experience rapid symptom relief with medium-dose corticosteroids, which are gradually tapered over several months. Treatment duration ranges from 3 to 36 months [11]. Non-steroidal anti-inflammatory drugs (NSAIDs), salicylates, and hydroxychloroquine have also been used to treat this disease [17]. Screening and treating for osteoporosis is also an important component of managing RS3PE due to the likelihood of long-term steroid therapy. Older adults are already at risk for low bone density, falls, and fractures, and bone health should be prioritized. Recurrence of RS3PE is not common and is associated with underlying malignancy [8].

## Conclusions

RS3PE should be considered in the differential diagnosis of an older adult with new onset inflammatory arthritis. It is a relatively new clinical condition, only first described in 1985. As the population ages and life expectancy increases, it is likely that clinicians will see an increase in the incidence of RS3PE. Understanding the clinical nuances and subtilties in distinguishing this condition from other inflammatory and crystalline arthropathies will lead to improved diagnostic accuracy and management. This patient was of advanced age, in his 90s, which may be a risk factor for this condition or could simply be a consequence of the increased prevalence of cancer with age. All patients with RS3PE require age-appropriate cancer screening, a detailed review of systems, and a thorough examination to uncover possible signs or symptoms of an undetected malignancy. More importantly, a patient’s comorbidities, life expectancy, goals of care, and exploration of “what matters most” (one of the pillars of age-friendly care) should guide clinicians on how best to balance additional diagnostic testing.
